# Link between neurodegeneration and trabecular meshwork injury in glaucomatous patients

**DOI:** 10.1186/s12886-017-0623-z

**Published:** 2017-11-28

**Authors:** Yong Zhang, Qinmei Yang, Feng Guo, Xia Chen, Lin Xie

**Affiliations:** Department of Ophthalmology, Daping Hospital & Research Institute of Surgery, Third Military Medical University, No.10, Changjiang Branch Road, Chongqing, 400042 People’s Republic of China

**Keywords:** Primary open angle glaucoma, Cataract, Neurodegeneration, Aqueous humour

## Abstract

**Background:**

Glaucoma is classified as a neurodegenerative disease. However, the biomarkers of neurodegeneration in the aqueous humour of primary open angle glaucoma (POAG) eyes have not been quantitatively examined yet. In this study, levels of neurodegeneration-related cytokines in the aqueous humour of POAG eyes were measured and compared with those of non-glaucoma (senile cataract) control eyes.

**Methods:**

This cross-sectional study included 24 patients (24 eyes) with POAG and 22 patients (22 eyes) with cataract. Aqueous humour samples were collected before the commencement of phacoemulsification surgery. The concentrations of brain-derived neurotrophic factor (BDNF), cathepsin D, myeloperoxidase (MPO), soluble intercellular adhesion molecule-1 (sICAM-1), soluble neural cell adhesion molecule (sNCAM), soluble vascular cell adhesion molecule-1 (sVCAM-1), and plasminogen activator inhibitor-1 (PAI-1) were measured using the Luminex suspension array technique. The clinical characteristics of the patients were also obtained for correlation analysis.

**Results:**

Compared with the cataract group, the levels of cathepsin D (*P* < 0.001), sNCAM (*P* < 0.001) and sVCAM-1 (*P* = 0.007) were significantly higher in the aqueous humour samples from POAG. The levels of BDNF, sICAM-1, MPO and PAI-1 did not differ among the groups. Mean deviation (MD) values measured by the Humphrey Visual Field Analyzer were significantly associated with levels of cathepsin D (*P* < 0.001; ρ= − 0.668), sICAM-1 (*P* = 0.003; ρ= − 0.579), sVCAM-1(*P* < 0.001; ρ= − 0.695), and PAI-1 (*P* = 0.007; ρ= − 0.533). The cytokines showed a positive correlation among each other (*P* < 0.0083).

**Conclusion:**

These data suggest that POAG patients had elevated levels of multiple biomarkers of neurodegeneration in the aqueous humour, and these elevated biomarkers may be related to trabecular meshwork injury.

**Trial registration:**

This study was registered in the Chinese Clinical Trial Registry (ChiCTR-OOC-16008516) on May 22, 2016.

## Background

Glaucoma is a group of optic neuropathy characterized by the degeneration of retinal ganglion cell (RGC) axons and somas [1]. It is the leading cause of irreversible blindness in the world, especially among the elderly. With the world population growing and the average lifespan increasing, glaucoma will affect more people. The number of people with glaucoma worldwide will increase to 76.0 million in 2020 and 111.8 million in 2040 [2].

Although glaucoma is defined primarily by optic nerve damage, neurodegeneration extends to the retina, lateral geniculate nucleus, and the occipital cortex [3, 4]. Recent research indicated that glaucoma destroy neurons through oxidative stress, impairment in axonal transport, neuroinflammation, and excitotoxicity [5]. As a neurodegenerative disorder, glaucoma even shares some similarities with other diseases in this category, such as Alzheimer’s disease (AD), Parkinson’s disease (PD), and multiple sclerosis (MS). These similarities include the selective loss of neuron populations, trans-synaptic degeneration in which the disease spreads from injured neurons to connected neurons, and common mechanisms of cell injury and death [6]. Glaucoma morbidity is higher among patients with AD and PD [7]; moreover optic nerves from AD patients are characterized by the loss of RGCs, the earliest dying cell in glaucoma [8].

Glaucoma can often be hard to notice until damage has already occurred [5]. In addition, lowering intraocular pressure (IOP) is currently the only way to delay the advancement of glaucoma. In view of the limitations of glaucoma diagnosis and therapy, some new potential biomarkers need to be found. Several studies have suggested that levels of neurodegeneration-related cytokines are altered in neurodegenerative diseases. For instance, elevated levels of cathepsin D in cerebrospinal fluid (CSF) was found in AD patients [9], and increased levels of sVCAM-1 and sICAM-1 in CSF was found in MS patients [10, 11]. The levels of these cytokines and possibly other biological factors may prove to be useful biomarkers of neurodegenerative disease.

Aqueous humour (AH) is the product of the ciliary body. It provides nutrition for the iris, cornea, lens, and trabecular meshwork [12]. In addition, it also exhibits anti-inflammatory potential, such as inhibiting neutrophil activation, preventing natural killer cells from lysing targets, suppress nitric oxide production by macrophages, and interfering with complement activation [13]. Altered cytokine levels in the AH have been observed in ocular disease, such as glaucoma [14], macular degeneration [15], and high myopic cataract [16]. Some investigators hypothesize that AH molecular alterations reflect glaucoma pathogenesis: the same events occur in the anterior chamber both at the level of the optic nerve and the central nervous system [17]. Therefore, the analysis of the AH can provide important information regarding the physiological and pathophysiological process in eyes. In POAG, the AH may play an important role in facilitating the migration of cytokines that stimulate the activity of human trabecular meshwork (HTM) cells [17, 18]. In addition, the AH contains several signalling molecules promoting synthesis, degradation, and modification of the extracellular HTM matrix [19]. Therefore, changes in the AH proteome may reflect cellular damage in HTM. However, a study in the literature reported that the neurodegenerative marker in the AH of POAG eyes is not adequate; thus, the present study measured biomarkers of neurodegeneration (BDNF, cathepsin D, sICAM-1, MPO, sNCAM, sVCAM-1, and PAI-1) in the AH from POAG patients.

## Methods

### Subjects and enrolment criteria

This study was approved by the Ethical Review Committee of Daping Hospital of the Third Military Medical University (No._47) and adhered to the provisions of the Declaration of Helsinki for research involving human subjects. We also registered in the Chinese Clinical Trial Registry (ChiCTR-OOC-16008516) on May 22, 2016. Each patient recruited in this study was given an explanation about the research and signed an informed consent for AH collection. Participants were recruited between June 2016 and December 2016. All patients were from a Chinese Han population. The axial length of these patients was measured using an IOL Master device (Carl Zeiss AG, Oberkochen, Germany), and the Mean deviation (MD) values was measured by the Humphrey Visual Field Analyzer (Carl Zeiss Meditec, Dublin, CA).

The diagnostic criteria of the POAG group were according to the following: (1) an open iridocorneal angle; (2) the characteristic appearance of glaucomatous optic neuropathy such as enlargement of the optic disc cup or focal thinning of the neuroretinal rim; (3) corresponding visual field defects tested; and (4) no evidence of secondary glaucoma.

The exclusion criteria for all groups included the following: (1) ocular inflammatory disease, systemic inflammatory, autoimmune, or pre-existing ocular disease (diabetic retinopathy, age-related macular degeneration and retinal vein/artery occlusion); (2) other neurodegeneration disease (Alzheimer’s disease, Parkinson disease or multiple sclerosis); (3) previous intraocular surgeries; (4) an AH sample less than 50 μL; (5) and incomplete data. These criteria were in compliance with the International Society for Geographical and Epidemiological Ophthalmology (ISGEO) classification of glaucoma in prevalence surveys by Foster et al. [20].

All POAG patients recruited were receiving IOP-lowering medication in the form of monotherapy or a combination of up to four of the following compounds: Brimatoprost, Tafluprost, Latanoprost (prostaglandin derivatives), Timolol (β-blocker), Brimonidine (α2-agonist), Dorzolamide, and Brinzolamide (carbonic anhydrase inhibitors).

### Collection of aqueous humour samples

All AH samples were collected under sterile conditions via an anterior chamber paracentesis before the commencement of phacoemulsification surgery. Patients were under general anaesthesia or local anaesthesia. Approximately 50–100 μL of the undiluted AH samples were collected by a single surgeon with a 30-gauge needle. Surgeries were performed between 9:00 am and 12:00 am. The AH samples were frozen and stored at −80 °C within 10 min until further analysis.

### Multiple immunoassay analyses

Concentrations of cytokine in AH were detected using multiplex bead-based immunoassays (Luminex, Merck, USA) with Human Neurodegenerative Disease Panel 3 (BDNF, cathepsin D, sICAM-1, MPO, sNCAM, sVCAM-1 and PAI-1). The assays were performed according to the manufacturer’s instructions. [21] Briefly, samples were thawed and centrifuged at 10000 ×g for 5 min to remove precipitates. A 25 μL aliquot of the AH was transferred to a 96 well pre-wet filter plate, and a part of each sample was placed into one of the capture microsphere multiplexes. Sample and capture microspheres were completely mixed and incubated at 4 °C for 18 h (protected from light). After two washes, multiplexed cocktails of biotinylated reporter antibodies were transferred and mixed. After incubation for 1 h at room temperature and two washes, multiplexes were developed using an excess of the solution of streptavidin plus phycoerythrin. The solution was mixed into each multiplex, after which it was incubated at room temperature for 30 min. Follow a washing step; a Luminex 200 Instrument (Luminex Corporation, TX, USA) was used for analysis and proprietary data analysis software (MILLIPLEX Analyst. Vision 5.1) was used for interpretation of the data.

### Statistical analysis

Data were analysed by using the SPSS for Windows, Version 17.0 (IBM-SPSS, Chicago, IL). The Kolmogorov-Smirnov test was used for the normality test. For comparisons of each pair of senile cataract and POAG groups, the differences in quantitative data including age, IOP, AL, and concentration of cytokines were calculated with the Mann-Whitney U test. Differences in categorical data including gender and eyes were determined using the Fisher’s exact probability test. Correlations among cytokines and correlations between cytokine concentrations and subjects’ demographic data (including age, IOP, mean deviation and glaucoma medications) were calculated using the Spearman’s correlation test. For the correction of multi-group comparisons, *P* values of 0.0083 for the Spearman’s correlation test were considered to be statistically significant at a level of 5% based on Bonferroni’s methods [22].

## Results

### Patient characteristics

AH samples were collected from 46 patients: 24 patients with POAG and 22 patients with cataract (non-glaucoma). The characteristics of patients, including age, gender, preoperative IOP, axial length (AL), mean deviation (MD), and glaucoma medications are summarized in Table 1 and Table 2. Preoperative IOP was higher in POAG eyes (22.22±7.40) than in cataract eyes (14.04±2.98), as calculated by a Mann-Whitney U test (*P* < 0.001).

**Table 1:** Patient characteristics

Characteristics	Cataract	POAG
Number of patients	22	24
Eye (left/right)	12/10	11/13
Sex (male/female)	10/12	10/14
Age, y		
Mean ± SD	65.59 ± 10.21	62.21 ± 9.32
Range	45–84	44–75
Preoperative IOP, mm Hg		
Mean ± SD	14.04 ± 2.98	22.22 ± 7.40**
Range	8.3–21.0	9.8–34.2
AL, mm		
Mean ± SD	24.56 ± 2.27	25.00 ± 2.26
Range	22.50–29.58	22.25–29.75
MD in Humphrey visual field analysis, dB		
Mean ± SD	Untested	−17.41 ± 8.98
Range	Untested	−31.02 to −0.94

POAG, primary open angle glaucoma, SD, standard deviation; IOP, intraocular pressure; AL, axial length, MD, mean deviation

**P < 0.01, calculated by a Mann-Whitney U test

**Table 2:** Glaucoma medications

Glaucoma medications,	Cataract	POAG
No. mean ± SD	0	2.5 ± 0.78
Range	0	1–4
β-blockers	0	22(92)
Prostaglandin analoguess	0	16(67)
Carbonic anhydrase inhibitors	0	12(50)
α2-agonist	0	10(42)

### Comparison of cytokines between POAG patients and cataract patients

The concentrations of the seven cytokines are shown in Table 3. Compared with the senile cataract group, the concentrations of cathepsin D, sNCAM and sVCAM-1 were significantly higher in AH samples from POAG (all *P* < 0.05). There were no significant differences in the levels of BDNF, sICAM-1, MPO, or PAI-1 between the two groups.

**Table 3:** Comparison of cytokine levels in the AH of eyes with POAG and cataract

Cytokine	POAG (pg/ml)	Cataract (pg/ml)	*P* value
BDNF	0.84 ± 0.13	0.89 ± 0.14	0.119
Cathepsin D	218,382.92 ± 32,671.62	178,882.82 ± 27,384.93	<0.001
sICAM-1	283.90 ± 188.16	236.46 ± 164.70	0.194
MPO	417.01 ± 600.22	290.11 ± 212.17	0.680
sNCAM	11,229.08 ± 2479.73	8391.14 ± 1717.89	<0.001
sVCAM-1	13,506.13 ± 8968.03	6930.14 ± 2581.25	0.007
PAI-1	544.45 ± 213.43	446.53 ± 220.17	0.141

POAG, primary open angle glaucoma; BDNF, brain-derived neurotrophic factor; sICAM, soluble intercellular adhesion molecule; MPO, myeloperoxidase; sNCAM, soluble neural cell adhesion molecule; sVCAM, soluble vascular cell adhesion molecule; PAI, plasminogen activator inhibitor

*P* values are calculated by Mann-Whitney U test

### Correlation analysis among cytokines in patients with POAG

The correlation analysis among cytokines in POAG patients is shown in Table 4. Statistical analysis revealed high correlations among sICAM-1, sNCAM, sVCAM-1 and PAI-1 (*P* < 0.0083 in all combinations). The level of sVCAM-1 were significantly associated with the concentration of cathepsin D (*P* = 0.002, ρ = 0.592). The concentration of PAI-1 was also correlated with MPO (*P* = 0.005, ρ = 0.551).

**Table 4:** Correlations among cytokines in POAG

ρ/*P* value	BDNF	Cat D	sICAM-1	MPO	sNCAM	sVCAM-1	PAI-1
BDNF	–	−0.103	−0.402	−0.192	−0.378	−0.412	−0.455
Cathepsin D	0.631	–	0.432	0.072	0.369	0.592	0.290
sICAM-1	0.052	0.035	–	0.425	0.743	0.737	0.751
MPO	0.368	0.740	0.038	–	0.312	0.269	0.551
sNCAM	0.069	0.076	0.000*	0.138	–	0.603	0.775
sVCAM-1	0.046	0.002*	0.000*	0.204	0.002*	–	0.620
PAI-1	0.025	0.169	0.000*	0.005*	0.000*	0.001*	–

POAG, primary open angle glaucoma; BDNF, brain-derived neurotrophic factor; Cat D,cathepsin D; sICAM, soluble intercellular adhesion molecule; MPO, myeloperoxidase; sNCAM, soluble neural cell adhesion molecule; sVCAM, soluble vascular cell adhesion molecule; PAI, plasminogen activator inhibitor

Correlation coefficient (ρ) and P values for each pair of cytokines are calculated by the Spearman’s correlation test

The *P* values are shown on the lower left side and ρ on the upper right side of the table

*Significance level at 5% (*P* < 0.0083), by Bonferroni correction for multiple comparisons

### Relationships of the subjects’ demographic data to cytokine concentrations in aqueous humour

**Fig. 1:**
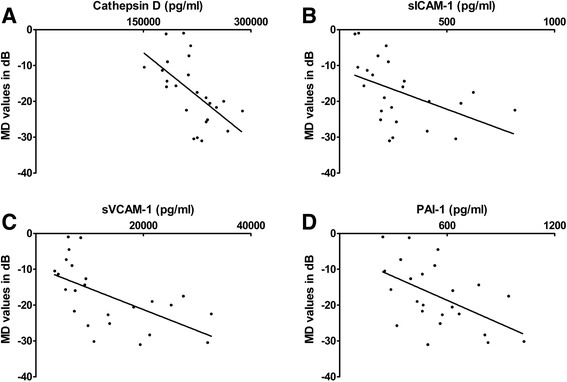
Relationships of MD (dB) to cytokine concentrations. The scatter grams showing the correlations between the MD in Humphrey Visual Field Analyzer and the levels of cathepsin D (**a**), sICAM-1 (**b**), sVCAM-1 (**c**) and PAI-1 (**d**) in AH of eyes with POAG. The x-axes represent the levels of cytokines, and the y-axes represent the MD values (dB).

Correlations between the levels of cytokines and clinical Variables in POAG are showed in Table 5. Statistical analysis reveals that age, IOP and glaucoma medications were not significantly correlated with the concentration of cytokines in AH of POAG patients. MD values measured by Humphrey Visual Field Analyzer was significantly associated with the levels of the cathepsin D (*P* < 0.001; ρ= − 0.668), sICAM-1 (*P* = 0.003; ρ= − 0.579), sVCAM-1(*P* < 0.001; ρ= − 0.695), and PAI-1 (*P* = 0.007; ρ= − 0.533) (Fig. 1).

**Table 5:** Correlations between the levels of cytokines and clinical variables

	Age (y)		IOP (mm Hg)		Glaucoma Medications(n)		MD (dB)	
	ρ	P	ρ	P	ρ	P	ρ	P
BDNF	0.102	0.635	−0.194	0.363	0.256	0.227	0.046	0.831
Cathepsin D	0.418	0.042	−0.146	0.496	0.115	0.594	−0.668	0.000*
sICAM-1	0.030	0.891	−0.130	0.543	−0.444	0.030	−0.579	0.003*
MPO	0.023	0.916	−0.036	0.868	−0.192	0.369	−0.340	0.104
sNCAM	−0.185	0.388	−0.041	0.848	−0.167	0.436	−0.426	0.038
sVCAM-1	0.277	0.190	0.124	0.563	−0.211	0.323	−0.695	0.000*
PAI-1	−0.216	0.311	−0.015	0.945	−0.176	0.411	−0.533	0.007*

Correlation coefficient (ρ) and P values are calculated by the Spearman’s correlation test

*Significance level at 5% (*P* < 0.0083), by Bonferroni correction for multiple comparisons

## Discussion

This study, which aimed to identify the biomarkers related to glaucoma has been difficult because the small volume of AH available for sampling. The multiplex bead immunoassay enabled us to measure multiple cytokine levels in a small sample. Thus, multiplex bead immunoassay can be a useful method for assessing biomarkers in AH sample. Moreover, it is a potent technology for introducing laboratory medicine into ophthalmology [23].

Using this technique, we demonstrated that levels of cathepsin D, sNCAM, and sVCAM-1 were significantly increased in POAG patients compared to the cataract group, while the levels of BDNF, sICAM-1, MPO, and PAI-1 were not differ among the groups. To the best of our knowledge, this study is the first to compare the concentrations of neurodegenerative biomarkers in the AH of patients with glaucoma vs. cataract.

Elevated levels of cathepsin D, sNCAM, and sVCAM-1 cannot be explained simply by an impaired blood-aqueous barrier (BAB). Increased local production in the anterior ocular segment may be another explanation. The alterations detected in the AH may be related to oxidative stress, mitochondrial alterations, apoptosis, tissue disaggregation, and neuronal damage [17]. Among them, oxidative stress has been recognized as a main pathogenic factor for POAG [24]. As age increases, the mitochondrial respiratory function decreases, which increases the production of reactive oxygen species (ROS) and free radicals in mitochondria [25]. When the levels of free radicals increase and the antioxidant defence is not sufficient, then oxidative stress may damage the HTM. Thus, these proteins whose AH levels are increased in POAG may reflect molecular and cellular damage in the HTM.

We measured the concentrations of three cell adhesion molecules (CAMs) including sICAM-1, sVCAM-1, and sNCAM. To date, we have not found any studies that indicate the presence of the CAM in AH of patients with POAG. CAMs are members of the immunoglobulin (Ig) superfamily that are involved in generating and maintaining cell connections and compose an extensive cell-cell and cell-matrix network [26]. Their presence in the AH indicated that the HTM had been severely damaged. These finding may explain the observation at the molecular level that the HTM undergoes progressive cell loss and cell disaggregation during POAG [27]. The expression of CAMs is induced by inflammatory cytokines [28] and might play a crucial role in inflammatory mechanisms [29].

In the present study, we found that levels of sNCAM and sVCAM-1 were significantly elevated in the AH of patients with POAG. VCAM-1 is an early marker of endothelial activation and dysfunction, leukocyte infiltration, and vascular remodelling [30]. Its expression can be induced by tumour necrosis factor-α (TNF-α) [31], and elevated concentrations of TNF-α have been found in AH of POAG eyes [32]. In the anterior ocular segment, VCAM-1 can be expressed by an HTM cell [33]. Moreover, some studies have found that the expression of VCAM-1 may be related to oxidative stress; the expression of VCAM-1 can be suppressed because of the inhibition of oxidative stress [34, 35]. The NCAM is a cell adhesion molecule that has been widely implicated in activating some signalling pathways, influencing cell migration, axonal outgrowth, and synaptogenesis [36]. Soluble forms of NCAM have been identified in blood, CSF, and neuronal cell culture media. Altered sNCAM levels in CSF have been observed in neurological disorders including AD [37], MS [38], schizophrenia [39], and bipolar disorder [40]. Evidence showed that sNCAM levels in blood plasma of patients may be used for the differential diagnosis of AD [41, 42]. Moreover, levels of low-molecular-weight forms of NCAM in the serum samples are correlated with the severity of dementia [43]. In the field with glaucoma, a significant increase in NCAM mRNA levels was detected by RT-PCR and Northern blots in cultured optic nerve head astrocytes within 6 h after exposure to elevated pressure [44]. Foets [45] found that the HTM can also express the NCAM, and may be important in the modelling of anterior ocular structures. The concentration of ICAM was also elevated, but there was no significant difference between the control group and the control group, which we thought was related to the sample size.

In this study, we also found that the elevated concentrations of cathepsin D in the AH of POAG. Cathepsin D is the main lysosomal aspartic protease that is expressed in all human cells. It is a protease that plays a crucial role in cell homeostasis since it is involved in both prosurvival (intralysosomal proteolysis of autophagy and endocytosed substrates) and prodeath (proteolysis of cytosolic substrates) processes [46]. On cultured cells, oxidative stress has been found to cause destabilization of lysosomal membranes by the peroxidation of membrane lipids [47]. In the anterior ocular segment, the generation of intralysosomal ROS induces lysosomal membrane permeabilization and the release of cathepsin D into the cytosol, leading to TM cell death [48]. Many altered neuronal proteins that hallmark neurodegenerative diseases (such as the amyloid, α-synuclein, and huntingtin) are physiologic substrates of cathepsin D and would abnormally accumulate if not efficiently be degraded by this enzyme [46]. Abnormally elevated levels of cathepsin D were reported in the CSF of AD patients. Moreover, cathepsin D plays a key role in the pathogenesis and progression of human neurodegenerative diseases. Although the exact pathophysiologic roles of cathepsin D in the AH of POAG patients have not been clarified, increased levels of cathepsin D in the AH of patients with POAG suggest a link between this protein and HTM injury.

We also provide evidence of a correlation between the levels of neurodegenerative biomarkers in the AH and the severity of visual field defects in POAG patients. The MD values were significantly associated with the levels of cathepsin D, sICAM-1, sVCAM-1, and PAI-1. However, a large sample size and long-term follow-up are needed to conclude that the cytokine levels in the AH truly reflect the severity of the visual field defect. In addition, statistical analysis reveals that age, IOP and glaucoma medications were not significantly correlated with the concentration of cytokines in the AH of POAG patients.

## Conclusions

In conclusion, the data demonstrated that cathepsin D, sNCAM, and sVCAM-1 levels were significantly increased in the AH of POAG patients compared with controls, and these elevated biomarkers may be related to trabecular meshwork injury.

## Abbreviations

AD: Alzheimer’s disease
AH: aqueous humour
AL: axial length
BAB: blood-aqueous barrier
BDNF: brain-derived neurotrophic factor
CSF: cerebrospinal fluid
IOP: intraocular pressure
MD: mean deviation
MPO: myeloperoxidase
MS: multiple sclerosis
OCT: Ocular Coherence Tomography
PAI: plasminogen activator inhibitor
PD: Parkinson’s disease
POAG: primary open-angle glaucoma
RGC: retinal ganglion cell
sICAM: soluble intercellular adhesion molecule
sNCAM: soluble neural cell adhesion molecule
sVCAM: soluble vascular cell adhesion molecule
